# Capillary Deposition of Complement C4d and C3d in Chinese Renal Allograft Biopsies

**DOI:** 10.1155/2015/397613

**Published:** 2015-03-04

**Authors:** Rong Lv, Wei Zhang, Fei Han, Guangjun Liu, Wenqing Xie, Jianghua Chen

**Affiliations:** ^1^Kidney Disease Center, The First Affiliated Hospital, College of Medicine, Zhejiang University, Hangzhou, Zhejiang 310003, China; ^2^Key Laboratory of Nephropathy, Hangzhou, Zhejiang 310003, China; ^3^Kidney Disease Immunology Laboratory, The Third Grade Laboratory, State Administration of Traditional Chinese Medicine, Hangzhou, Zhejiang 310003, China; ^4^Key Laboratory of Multiple Organ Transplantation, Ministry of Health, Hangzhou, Zhejiang 310003, China

## Abstract

*Background*. C3d is a product of both the classic and the alternative complement cascades; however, few studies have addressed the role of C3d in renal biopsies and its relationship with long-term graft survival rate is not very clear. *Methods*. 94 patients with biopsy-proven acute rejection episodes were included in the study. We investigated the associations between histological findings, clinical examinations, and outcome. *Results*. The overall prevalence for C4dPTC and C3dPTC was 42.6% and 29.8%. There was a significant association between C3dPTC and C4dPTC (*P* < 0.001). C3dPTC and C4dPTC were related with histological types (*P* = 0.024 and *P* < 0.001, resp.). The long-term survival rate for C4dPTC positive transplants was lower than that of C4dPTC negative transplants, but it was not statistic significant in our study (*P* = 0.150). The survival rate of C3dPTC positive group was much lower than the negative group (*P* = 0.014). Patients with double positives for C4dPTC and C3dPTC exhibited the lowest survival rate significantly different from those of the C3dPTC only and C4dPTC only groups (*P* = 0.01 and *P* = 0.0037). *Conclusions*. This longitudinal cohort study has demonstrated that C3d deposition in the PTC was closely related to renal dysfunction and pathological changes.

## 1. Introduction

Renal transplantation has been so far the best way to treat end-stage renal disease (ESRD). However, doctors and patients are still facing many challenges during the implementation of the clinical procedure, among which renal rejection has always been a major concern. Acute rejection (AR), the leading cause for renal allograft dysfunction, has been classified according to the Banff-97 criteria [1]. Based on data from modern series, the incidence of antibody-mediated acute rejection (AbAR) is significant, reported between 0 and 8% in renal allograft recipients in large centers. Within the past decade, reports have emerged regarding the usefulness of peritubular capillary (PTC) staining for C4d as a marker of AbAR. PTC deposition of the complement fragment C4d identifies patient who has developed acute humoral rejection and is used as a strong predictor for long-term graft survival rate [2–4]. Although C4dPTC has been the focus of many studies in adult renal transplantation, C3dPTC has received much less attention. To date, there have been only a limited number of studies on C3d staining in renal allograft [5, 6]. C3d is a product of both the classic and the alternative complement cascade. Sund et al. [5] reported that, in 37 protocol biopsies taken a median of 7 days after transplantation, they found 11 C4d positive with concurrent C3d deposition in 3. Graft loss within 2 months occurred in two of the 3 C3d-positive cases, while only one C3d-negative graft was lost. Another research group examined C3dPTC in adults with AR biopsies taken during the first year after transplantation. Comparing outcomes of patients with C3dPTC positive and C3dPTC negative [6], they found that patients with C3d-positive rejection were significantly more likely to have delayed graft function and also had a higher rate of graft loss than those with C3d-negative rejection (23% versus 7%). But the sample number was too small to allow for significant analysis of survival among the different combination of C3dPTC and C4dPTC groups. Moreover, the short follow-up time in the existing studies failed to reflect the relationship between C3dPTC and long-term survival rate of the graft. The purpose of this study was to determine the relationship between C4d and C3d and the effect of peritubular capillary deposition of C4d and C3d with rejection type, and with graft long-term survival rate in Chinese renal recipients.

## 2. Materials and Methods

### 2.1. Patients and Biopsies

This retrospective study analyzed histologic findings and clinical data in renal allograft recipients at the Nephrology Center of the First Affiliated Hospital of Zhejiang University. 94 patients with biopsy-proven AR episodes occurring during the first year after transplantation between 1999 and 2003 were included in the study. Multitransplant and multiorgan transplant patients were excluded. Clinical and laboratory data and follow-up data for subjects experiencing acute rejection were extracted from their medical records in our center, including their age, gender, primary disease, posttransplantation day at biopsy, serum levels of creatinine and panel-reactive antibody (PRA) level at the time of biopsy, type of immunosuppressive drugs used, human leukocyte antigen (HLA) mismatch, cold and warm ischemic times, serum PRA level and dialysis duration before transplantation, serum creatinine at 3, 6, and 12 month after transplantation, acute rejection episodes, and graft outcome. The follow-up data were all available in transplant database. The end-point was November 2013 or the day of graft loss, the mean follow-up was 2856 ± 1703 days.

### 2.2. Immunosuppressant

All the patients received triple therapy of cyclosporine (CsA) or tscrolimus (FK506)+steroid+MMF. Methylprednisolone (MP) was administered intravenously at a dose of 500 mg during the transplantation and on days 1 and 2 and of 250 mg on day 3 after the operation. It was switched to oral prednisolone at a dose of 80 mg/d and tapered to a maintenance dose of 10 mg/d after three months. The daily CsA (FK506) dose was initiated at an oral dose of 6-7 mg/kg (0.1 mg/kg) until the value of serum creatinine declined to 250 umol/L and adjusted according to the blood levels of CsA (FK506), which were maintained at 250–300 ng/mL (6–8 ng/mL) after 3 months. MMF was given at a dose of 1.0–2.0 g/d. Induction therapy with antithymocyte globulin (ATG) or anti-CD3 monoclonal antibodies (OKT3) was not routinely given. Rejection episodes were treated with a large dose of MP (4–6 mg/kg) for 3–5 days, and, to some patients with steroid-resistant rejection, intravenous ATG (100 mg/d) or OKT3 (5 mg/d) was used for 5–7 days. And two patients received double-filtration plasmapheresis (DFPP).

### 2.3. C3d Staining

Rabbit-originated polyclonal antibody (purchased from Biomedical Corporation, Vienna, Austria) was used to detect C3d in paraffin-embedded tissue. Four um thick sections were routinely deparaffinized and the endogenous peroxidase activity was blocked by 3% hydrogen peroxide. Antigen retrieval was achieved by pressure-cooking for 10 min at 1 bar in citrate-buffer (pH 6.0). Sections were firstly incubated overnight at 4°C with C3dAb (dilution 1 : 1 : 500 Dakopatts A/S, DK-2600 Glostrup, Denmark) and washed in PBS for three times, again, followed by incubating 10 min with a secondary antibody, IgG antibody- (Fab-) HRP polymer which was used according to the manufacturer's protocol. Tissues then were stained for 10 min with fresh diaminobenzidin (DBA) solution.

C4d staining was routinely performed on all renal allograft biopsies at our center during the study period. Biopsies were assessed for C4d and C3d staining by two different researchers (double blind). The results were considered C4d-positive or C3d-positive if more than one of their biopsies exhibited circumferential staining for over 25% of the peritubular capillaries.

### 2.4. Statistical Analyses

Descriptive statistical values are presented as mean ± SE or as medians with 25th and 75th percentile values, depending on the underlying distribution. Continuous variables were compared by the *t*-test or the Wilcoxon rank-sum test where appropriate. Categorical variables were compared by the *χ* ^2^ test. A *P* value of <0.05 was considered significant. Graft survival rates were estimated by using the Kaplan-Meier method, and survival curves were compared by the log-rank test. The Cox proportional-hazards model was used to identify important predictors of the time to graft loss.

## 3. Results

### 3.1. The Prevalence of C4dPTC and C3dPTC

**Figure 1:**
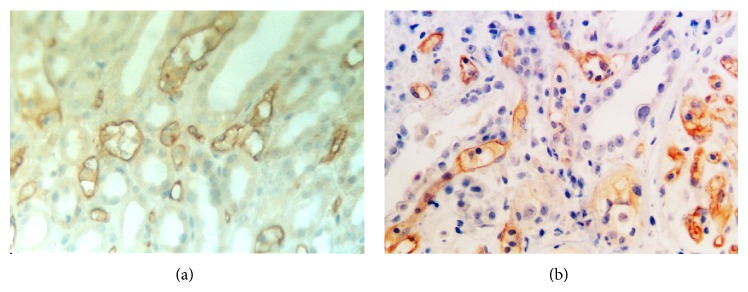
(a) C4dPTC positive ×400. (b) C3dPTC positive ×400.

We obtained a total of 94 biopsy samples from patients with active AR episodes and stained them for C4d and C3d. Typical staining patterns are shown in Figure 1, where C4d and C3d both exhibit a linear structure along the entire circumference of the peritubular capillaries. We defined a positive case by the appearance of such characteristic staining in more than 2 samples from the patient, while excluding C4d or C3d deposition in the glomerulus. The overall prevalence for C4dPTC and C3dPTC calculated using such criteria was 42.6% and 29.8%, respectively. Among them, 47 biopsies are C3d−C4d−, 19 biopsies are C3d−C4d+, 7 are C3d+C4d−, and 21 are C3d+C4d+. The association study revealed a significant correlation between C3dPTC and C4dPTC (*P* < 0.001).

### 3.2. Demographic Data

Patient demographics are summarized in Table 1. The subgroups in C3dPTC or C4dPTC have no statistical differences in gender, age, warm ischemic time, cold ischemic time, dialysis duration, and HLA mismatch. But they are all related to the pretransplant PRA (*P* = 0.048 and *P* = 0.031 resp.).

### 3.3. Histological Findings

Renal biopsies were divided into three portions, for light microscopic, electron-microscopic, and immunohistochemical analysis. Tissues were fixed in 10% buffered formalin and embedded in paraffin, cut in 4 um thick sections and stained with hematoxylin-eosin, periodic acid-Schiff stain, and Masson's trichrome. The Banff 97 classification for the diagnosis was used. For 30 patients, biopsies demonstrated vascular rejection (IIA), for 32 patients, biopsies showed cellular rejection (IA and IB), and 32 biopsies showed borderline with creatinine elevated 20% than the baseline in clinical.

The histological findings in different subgroups of C3dPTC and C4dPTC were summarized in Tables 2 and 3. C3dPTC and C4dPTC were related with histological types (*P* = 0.024 and *P* < 0.001, resp.). Like C4dPTC staining, C3dPTC positive can be seen in all kinds of rejections, but it is commonly seen in the vascular rejection. C3dPTC, but not C4dPTC, was associated with tubulitis (*P* = 0.049), while C4dPTC was much more related to glomerulitis (*P* = 0.034). They were all associated with intimal arteritis (*P* < 0.001 and 0.001, resp.), but the differences in other pathological features, interstitial inflammation, mesangial proliferation, and immunofluorescence stain of C3 and C4, were not significant between the C4dPTC (C3dPTC) positive and negative groups. In all biopsies, 16 were found to display chronic allograft nephropathy changes, but the difference between C3dPTC subgroups and C4dPTC subgroups was not significant.

### 3.4. Clinical Correlates

Acute rejection developed on average about 3 months after transplantation in C4dPTC positive and C3dPTC positive groups, which were much later than the negative groups. The highest and the lowest creatinine levels during the acute rejection were also significantly higher in C4dPTC positive and C3dPTC positive groups while compared to their negative groups. 19 patients had graft loss during the acute rejection, but there was no significance between the C3dPTC positive and negative groups or C4dPTC positive and negative groups. The creatinine level was different in one year after transplant between C4dPTC+ and C4dPTC− groups whereas that in C3dPTC positive and negative groups was different in only 6 months after transplant (Table 4).

A total of 89 serum samples taken at the time of biopsy were analyzed for PRA (Table 5). 32.4% of the C4d-positive biopsies were associated with the presence of PRA. PRA positive was also noted in 11.5% C4d-negative biopsies (*P* = 0.03). 44% C3d positive biopsies were related to the PRA, while it also appeared in 10.9% of the C3d negative group. 55.6% of C4d and C3d double positive biopsies could find PRA, which was much higher than the C4d negative and C3d negative group (*P* = 0.001).

**Figure 2:**
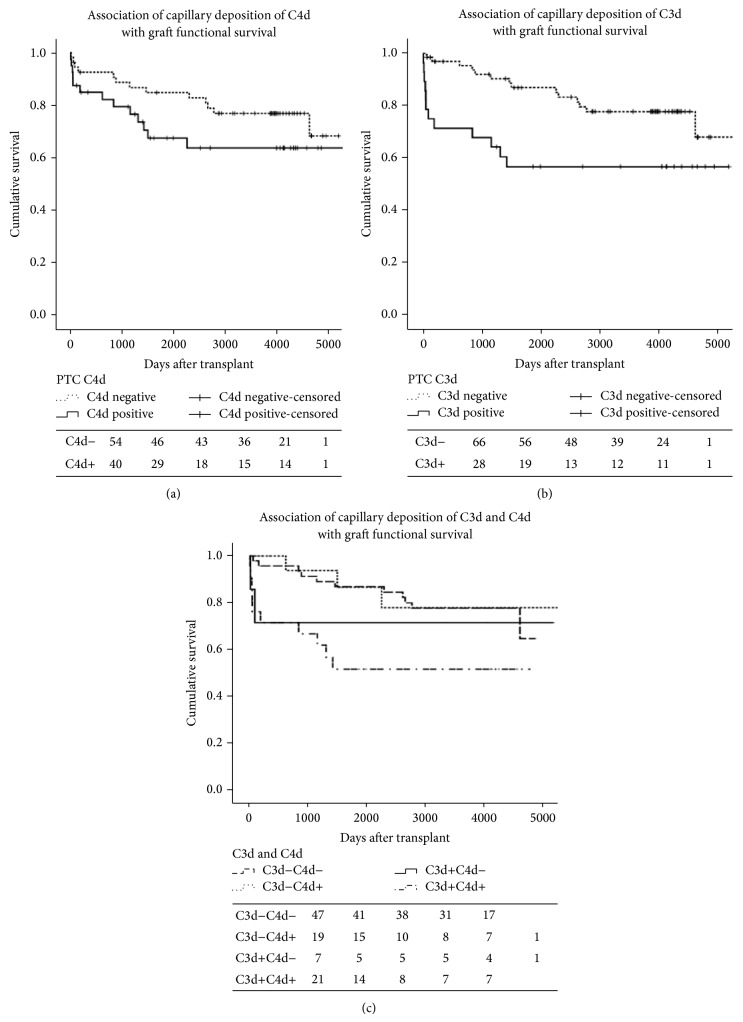
(a) Association of capillary deposition of C4d with graft functional survival (C4d negative versus C4d positive, log rank 2.077, *P* = 0.150). (b) Association of capillary deposition of C3d with graft functional survival (C3d negative versus C3d positive, log rank 6.043, *P* = 0.014). (c) Association of capillary deposition of C3d and C4d with graft functional survival (C3d−C4d− versus C3d+C4d+, log rank 6.654, *P* = 0.01; C3d−C4d+ versus C3d+C4d+, log rank 4,345, *P* = 0.0037; others have no significance).

The long-term survival rate of C4dPTC positive transplants was lower than that of C4dPTC negative transplants, but it was not statistically significant in our study (log rank 2.077, *P* = 0.150). C3dPTC had great impact on graft functional survival; the survival rate of C3dPTC positive group was much lower than the negative group (log rank 6.043, *P* = 0.014), while, combined C4dPTC with C3dPTC, the survival rate was lowest in C3dPTC+C4dPTC+ group, and it has reached statistical significance when compared to the C3dPTC−C4dPTC− or the C3dPTC−C4dPTC+ group (*P* = 0.01 and *P* = 0.0037, resp.) (Figures 2(a), 2(b), and 2(c)).

The Cox proportional-hazards model was used to identify important predictors of the time to graft loss (Table 6). The variables included in multivariate analyses were patients' age, patient gender, dialysis duration, pre- and posttransplant PRA level, number of HLA mismatch, cold and warm ischemic time, C4dPTC, C3dPTC, and histological parameters. It shows that posttransplant PRA and C3dPTC were the independent risk factors for the graft functional survival with the odds ratio of 6.797 and 9.210, respectively (*P* = 0.019 and *P* = 0.025).

## 4. Discussion

This study analyzed the prevalence of C4dPTC and C3dPTC retrospectively in Chinese renal transplants allograft biopsies and investigated the possible association of these complement split products with histologic features and clinical outcome. To our knowledge, this is the most extensive longitudinal cohort study of adult kidney transplant recipients reported to date, as measured by the follow-up time of patients.

Many studies have suggested acute rejection as an important factor causing chronic rejection and renal allograft failure. But the influence of AR has been considered more complex than expected [7]. Within the past decades, reports have emerged regarding the usefulness of peritubular capillary staining for C4d as a marker of AbAR. C4d capillary staining may be correlated with poor outcome and requirement for more aggressive immunosuppressive therapy [4]. In our study, the prevalence of C4dPTC is 42.6%, and it is coincided with what had been reported in other studies [8, 9]. Our study demonstrated that the long survival rate of C4dPTC positive transplants was not significantly worse than that of C4dPTC negative transplants, likely due to the aggressive treatment with antithymocyte globulin or OKT3.

C3d is the final split product of C3, which is the central component of the complement system. C3d is a stable marker of complement activation that binds covalently to cell surfaces, so it can persist for a long time in the tissue. It is a ligand of complement receptor 2 (CR2) on B lymphocyte. The interaction between C3d and CR2 is a key aspect of complement immune system activation and is a component in a link between innate and adaptive immunities [10, 11]. In this study, we showed that C3dPTC positive staining is related to the type of acute rejection episodes and is an indicator of poor prognosis for long-term survival rate. C3dPTC was similar to C4dPTC, as they were both observed with comparable frequencies in AR biopsies. In this regard, C3dPTC can also be used as a marker for AbAR. A meaningful comparison among the four subgroups and the association with long-term survival rate shows that the group of C3d+C4d+ had the worst outcome. Although we cannot claim that C3dPTC is a more sensitive marker for prognosis, as more aggressive treatment was used for C4dPTC positive rejection episodes, this study suggests that C3d deposition in PTC is a useful biomarker for prognosis. However, C3dPTC is indeed different from C4dPTC, as all combinations of C3d and C4d deposition are present in our study (C3d−C4d−; C3d+C4d−; C3d−C4d+; C3d+C4d+). In biopsies where only C3d was found indicating the existence of possible amplification loops that only augments C3, but not C4 deposition under the influence of certain risk factors, the complement cascade is initiated through the classic, alternative, and lectin pathways. The classic pathway is antibody mediated, while the lectin and alternative pathways are triggered by distinct carbohydrate or lipid patterns on microbes or host cells. Whether the C3dPTC is activated by the alternative or the lectin pathway to some extent is at present unclear. In our paper, we just found the phenomenon of the C3dPTC with the worse outcome. Further research should be made to discuss this problem.

Rejection and graft survival are determined by many risk factors. Besides the immunosuppression regimen and transplant center effects, age, sex, dialysis duration, PRA, and rejection type, all have been proposed as high-risk factors. As C3dPTC is related to AR episodes and graft outcomes, we analyzed the relationship between C3dPTC and these factors. We were surprised to find that PRA, tubulitis, intimal arteritis, and rejection onset time were all associated with C3dPTC.

The product of DSA is an indicator for poor prognosis [12] and humoral components can speed up the development of chronic transplant nephropathy [13–16]. Unfortunately we carried out the DSA test 6 years ago in our hospital, so many patients lacked the data of DSA and we did not discuss the correlation between DSA and the C3d deposition in this paper. It is well known that recipients with high PRA levels show high risk of delayed graft function, AR, or even kidney loss [17]. Previous researches found that there was a significant degree of concordance between the posttransplant PRA and DSA [17]. Our study showed that the PRA levels differed significantly before and after transplant in C3dPTC-positive and -negative groups. We speculate that C3dPTC may correlate with DSA as well, which will be addressed in greater detail in our future study.

In all Banff scored lesions, interstitial inflammation and tubulitis alone have the greatest likelihood of reversibility and good graft outcome. Intimal arteritis is less responsive to steroid treatment and may require potent antibody therapy, especially for transmural arteritis and/or arterial fibrinoid necrosis [18–20]. In our study, C3dPTC positive was significantly associated with tubulitis and Intimal arteritis. Therefore, a positive C3d staining alone without evidence of histologic features of AMR should be interpreted with caution.

In our previous study, we discovered that early complete reversal of AR had no significant effect on the long-term outcome [21]. Another group found the similar result, and showed that later AR had poor clinical outcome and was often refractory to antirejection therapy [22]. Interestingly, in this retrospective study, we discovered that most C3dPTC positive AR took place after 3 months after transplantation.

The Cox proportional hazard models show that C3d is an independent risk factor for graft long-term survival rate. The way in which local deposition of C3d accelerates renal allograft rejection is not yet solved. Due to the significance of the C3d-CR2 interaction and its role in increasing B-cell sensitivity, extensive research has been performed trying to address the nature of the interaction, as well as to identify approaches for the design of new therapeutics and vaccines. Clinical renal biopsy studies have shown that there was a marked upregulation of C3 and C4 gene expression in a variety of inflammatory conditions [23, 24]. This is especially pronounced in rejecting kidney allografts [25]. In renal allografts, it has been demonstrated that there was defective T-cell priming in the absence of local synthesis of C3 [26], suggesting that locally produced C3 is more crucial for acute cellular rejection than the circulating host derived C3. Current evidence indicates that defective local synthesis of C3 can both reduce tissue injury and lower the antidonor T-cell response, therefore substantially increasing graft survival rate. Further studies are needed to investigate the relationship between the locally deposited C3d and rejection.

In summary, this longitudinal cohort study has demonstrated that C3d deposition in the PTC was closely related to renal dysfunction and pathological changes. C3dPTC is also an indicator for poor prognosis, which can be used in clinical practice. Further investigation of C3d and C4d levels should be considered for potential use in routine patient care, clinical research, and testing of potential novel therapeutic agents.

## Figures and Tables

**Table 1:** Patient population characteristics.

	C4d+/C4d− (*n* = 40/54)	*P*	C3d+/C3d− (*n* = 28/66)	*P*
Gender (*n* female)	13/15	0.654	9/19	0.807
Age	37.65 ± 10.21/37.50 ± 10.96	0.946	37.17 ± 10.80/38.50 ± 10.22	0.579
WIT (min)	6.74 ± 1.71/6.70 ± 1.95	0.909	7.04 ± 1.87/6.58 ± 1.87	0.280
CIT (min)	421.03 ± 141.03/441.70 ± 104.06	0.442	419.19 ± 142.37/438.95 ± 110.90	0.489
Dialysis duration	6.01 ± 12.54/4.19 ± 4.72	0.333	4.47 ± 8.10/5.13 ± 9.14	0.653
HLA mismatch	4.23 ± 0.93/3.72 ± 1.41	0.266	4.29 ± 1.11/3.83 ± 1.27	0.404
PRA (pre) >10%	15/7	0.048	11/11	0.031

WIT: warm ischemic time; CIT: cold ischemic time.

**Table 2:** The expression of C4d and C3d in different rejection type.

	Vascular rejection	Cellular rejection	Borderline	*χ* ^2^	*P*
C4d+/C4d−	23/7	12/20	5/27	24.1	<0.001
C3d+/C3d−	15/15	8/24	5/27	9.28	0.01

**Table 3:** The histological findings in different groups.

	C4d+/C4d− (*n* = 40/54)	*P*	C3d+/C3d− (*n* = 28/66)	*P*
Tubulitis	30/47	0.110	23/47	0.049
Interstitial inflammation	25/30	0.322	15/40	0.342
Glomerulitis	15/10	0.034	9/16	0.292
Intimal arteritis	22/3	<0.001	14/11	0.001
Mesangial proliferation	25/30	0.532	15/40	0.648
C3	7/8	0.569	5/10	0.757
C4	2/1	0.302	2/3	0.183
CAN	7/9	0.915	6/10	0.550

CAN: chronic allograft nephropathy.

**Table 4:** The clinical manifestation in different groups.

	C4d+/C4d− (*n* = 40/54)	*P*	C3d+/C3d− (*n* = 28/66)	*P*
AR time	106.93 ± 128.25/53.17 ± 73.48	0.021	109.32 ± 125.54/62.06 ± 90.04	0.049
Top cr	367.72 ± 232.26/252.98 ± 191.70	0.014	422.44 ± 284.23/251.43 ± 158.02	0.006
Bottom cr	171.83 ± 97.99/133.43 ± 34.25	0.029	188.88 ± 111.31/134.25 ± 37.59	0.027
Graft loss	5/14	0.127	6/13	0.848
3 m cr	129.22 ± 36.29/141.20 ± 86.62	0.454	158.24 ± 131.08/129.24 ± 31.95	0.106
6 m cr	139.44 ± 51.95/136.52 ± 74.50	0.834	164.74 ± 112.59/128.52 ± 37.20	0.029
1 y cr	158.90 ± 107.84/123.54 ± 43.65	0.045	140.45 ± 53.80/136.39 ± 83.75	0.84

Graft loss: this means that the graft did not recover the function during the acute rejection.

**Table 5:** Correlation of PTC C4d and C3d staining with PRA at the time of biopsy.

PTC staining	PRA+	PRA−	*P*
C4d+/C4d−	12/6	25/46	0.03
C3d+/C3d−	11/7	14/57	0.001
C4d+C3d+/C4d+C3d−	10/2	8/17	0.001
C4d−C3d+/C4d−C3d−	1/5	6/40	

**Table 6:** Clinical determinant of long-term graft functional survival rates (Cox proportional hazard models).

Variable	Odds ratio (95% confidence interval)	*P* value
PRA^*^	6.791 (1.377–33.49)	0.019
C3d ptc	9.210 (1.319–64.289)	0.025

^*^PRA: it represents the PRA after transplantation at the time of biopsy.

## References

[1] Racusen L. C., Solesz K., Colvin R. B. (1999). The Banff 97 working classification of renal allograft pathology. *Kidney International*.

[2] Feucht H. E., Schneeberger H., Hillebrand G. (1993). Capillary deposition of C4d complement fragment and early renal graft loss. *Kidney International*.

[3] Regele H., Böhmig G. A., Habicht A. (2002). Capillary deposition of complement split product C4d in renal allografts is associated with basement membrane injury in peritubular and glomerular capillaries: a contribution of humoral immunity to chronic allograft rejection. *Journal of the American Society of Nephrology*.

[4] Collins A. B., Schneeberger E. E., Pascual M. A. (1999). Complement activation in acute humoral renal allograft rejection: diagnostic significance of C4d deposits in peritubular capillaries. *Journal of the American Society of Nephrology*.

[5] Sund S., Hovig T., Reisæter A. V., Scott H., Bentdal Ø., Mollnes T. E. (2003). Complement activation in early protocol kidney graft biopsies after living-donor transplantation. *Transplantation*.

[6] Kuypers D. R. J., Lerut E., Evenepoel P., Maes B., Vanrenterghem Y., van Damme B. (2003). C3d deposition in peritubular capillaries indicates a variant of acute renal allograft rejection characterized by a worse clinical outcome. *Transplantation*.

[7] Vanrenterghem Y. (2000). Impact of acute rejection on the long-term outcome after renal transplantation. *Graft*.

[8] Nickeleit V., Zeiler M., Gudat F., Thiel G., Mihatsch M. J. (2002). Detection of the complement degradation product C4d in renal allografts: diagnostic and therapeutic implications. *Journal of the American Society of Nephrology*.

[9] Mauiyyedi S., Crespo M., Bernard Collins A. (2002). Acute humoral rejection in kidney transplantation: II. Morphology, immunopathology, and pathologic classification. *Journal of the American Society of Nephrology*.

[10] Toapanta F. R., Ross T. M. (2006). Complement-mediated activation of the adaptive immune responses. *Immunologic Research*.

[11] Sahu A., Lambris J. D. (2001). Structure and biology of complement protein C3, a connecting link between innate and acquired immunity. *Immunological Reviews*.

[12] Everly M. J., Everly J. J., Arend L. J. (2009). Reducing de novo donor-specific antibody levels during acute rejection diminishes renal allograft loss. *The American Journal of Transplantation*.

[13] Wiebe C., Gibson I. W., Blydt-Hansen T. D. (2012). Evolution and clinical pathologic correlations of de novo donor-specific HLA antibody post kidney transplant. *The American Journal of Transplantation*.

[14] Hidalgo L. G., Campbell P. M., Sis B. (2009). De novo donor-specific antibody at the time of kidney transplant biopsy associates with microvascular pathology and late graft failure. *American Journal of Transplantation*.

[15] Kimball P. M., Baker M. A., Wagner M. B., King A. (2011). Surveillance of alloantibodies after transplantation identifies the risk of chronic rejection. *Kidney International*.

[16] Thammanichanond D., Ingsathit A., Mongkolsuk T. (2012). Pre-transplant donor specific antibody and its clinical significance in kidney transplantation. *Asian Pacific Journal of Allergy and Immunology*.

[17] Kerman R. H., Orosz C. G., Lorber M. I. (1997). Clinical relevance of anti-HLA antibodies pre and post transplant. *The American Journal of the Medical Sciences*.

[18] Solez K., Colvin R. B., Racusen L. C. (2007). Banff '05 meeting report: differential diagnosis of chronic allograft injury and elimination of chronic allograft nephropathy (‘CAN’). *American Journal of Transplantation*.

[19] Bhowmik D. M., Dinda A. K., Mahanta P., Agarwal S. K. (2010). The evolution of the Banff classification schema for diagnosing renal allograft rejection and its implications for clinicians. *Indian Journal of Nephrology*.

[20] Elshafie M., Furness P. N. (2012). Identification of lesions indicating rejection in kidney transplant biopsies: tubulitis is severely under-detected by conventional microscopy. *Nephrology Dialysis Transplantation*.

[21] Wu J.-Y., Chen J.-H., Wang Y.-M. (2003). Completely reversed acute rejection episodes do not influence the long-term renal allograft survival. *Zhonghua Yi Xue Za Zhi*.

[22] Madden R. L., Mulhern J. G., Benedetto B. J. (2000). Completely reversed acute rejection is not a significant risk factor for the development of chronic rejection in renal allograft recipients. *Transplant International*.

[23] Welch T. R., Beischel L. S., Witte D. P. (1993). Differential expression of complement C3 and C4 in the human kidney. *The Journal of Clinical Investigation*.

[24] Sacks S. H., Zhou W., Andrews P. A., Hartley B. (1993). Endogenous complement C3 synthesis in immune complex nephritis. *The Lancet*.

[25] Andrews P. A., Pani A., Zhou W., Sacks S. H. (1994). Local transcription of complement C3 in human allograft rejection: evidence for a pathogenic role and correlation to histology and outcome. *Transplantation*.

[26] Pratt J. R., Basheer S. A., Sacks S. H. (2002). Local synthesis of complement component C3 regulates acute renal transplant rejection. *Nature Medicine*.

